# Biodistribution and radiation dosimetry of ^124^I-mIBG in adult patients with neural crest tumours and extrapolation to paediatric models

**DOI:** 10.1186/s40658-023-00604-0

**Published:** 2024-01-03

**Authors:** Alexandros Moraitis, Walter Jentzen, Gloria Reiter, Jochen Schmitz, Thorsten Dirk Pöppel, Manuel Weber, Ken Herrmann, Wolfgang Peter Fendler, Pedro Fragoso Costa, Andreas Bockisch, David Kersting

**Affiliations:** 1 Department of Nuclear Medicine, West German Cancer Center (WTZ), University Hospital Essen, University of Duisburg-Essen, Hufelandstrasse 55, 45147 Essen, Germany; 2 German Cancer Consortium (DKTK), Partner Site University Hospital Essen, Essen, Germany

**Keywords:** Dosimetry, ^124^I-mIBG, PET, Effective dose, Neural crest tumour

## Abstract

**Aim:**

Positron emission tomography (PET) using ^124^I-mIBG has been established for imaging and pretherapeutic dosimetry. Here, we report the first systematic analysis of the biodistribution and radiation dosimetry of ^124^I-mIBG in patients with neural crest tumours and project the results to paediatric patient models.

**Methods:**

Adult patients with neural crest tumours who underwent sequential ^124^I-mIBG PET were included in this retrospective single-center analysis. PET data were acquired 4, 24, 48, and/or 120 h after administration of a mean of 43 MBq ^124^I-mIBG. Whole-body counting and blood sampling were performed at 2, 4, 24, 48 and 120 h after administration. Absorbed organ dose and effective dose coefficients were estimated in OLINDA/EXM 2.2 according to the MIRD formalism. Extrapolation to paediatric models was performed based on mass-fraction scaling of the organ-specific residence times. Biodistribution data for adults were also projected to ^123^I-mIBG and ^131^I-mIBG.

**Results:**

Twenty-one patients (11 females, 10 males) were evaluated. For adults, the organs exposed to the highest dose per unit administered activity were urinary bladder (1.54 ± 0.40 mGy/MBq), salivary glands (0.77 ± 0.28 mGy/MBq) and liver (0.65 ± 0.22 mGy/MBq). Mean effective dose coefficient for adults was 0.25 ± 0.04 mSv/MBq (male: 0.24 ± 0.03 mSv/MBq, female: 0.26 ± 0.06 mSv/MBq), and increased gradually to 0.29, 0.44, 0.69, 1.21, and 2.94 mSv/MBq for the 15-, 10-, 5-, 1-years-old, and newborn paediatric reference patients. Projected mean effective dose coefficients for ^123^I-mIBG and ^131^I-mIBG for adults were 0.014 ± 0.002 mSv/MBq and 0.18 ± 0.04 mSv/MBq, respectively.

**Conclusion:**

PET-based derived radiation dosimetry data for ^124^I-mIBG from this study agreed well with historical projected data from ICRP 53. The effective dose coefficients presented here may aid in guidance for establishing weight-based activity administration protocols.

**Supplementary Information:**

The online version contains supplementary material available at 10.1186/s40658-023-00604-0.

## Introduction

Meta-iodobenzylguanidine (mIBG) is a synthetic analogue of the adrenergic neurotransmitter norepinephrine and is accumulated and retained in sympathicomimetic cells [1]. Tumours deriving from neuronal crests such as malignant pheochromocytoma, neuroblastoma, paraganglioma and some neuroendocrine tumours from the midgut have shown to accumulate mIBG with reported sensitivities up to 88% and specificities ranging from 80 to 100% [2, 3].

Because of its high specificity, radioiodinated mIBG has long been used in theranostics of patients with neuronal crest tumours. The application of the gamma-emitting ^123^I-mIBG is an established approach in staging and monitoring treatment response, whereas the beta-emitting ^131^I-mIBG can be used for both diagnosis and treatment [4, 5]. Of paramount importance, ^131^I-mIBG was approved in 2018 by the Food and Drug Administration for the treatment of unresectable, locally advanced or metastatic pheochromocytoma and paraganglioma, and as a consequence, the number of patients benefitting from this treatment option is expected to increase. However, in extensively treated patients, ^131^I-mIBG therapy can cause myelosuppression, which might necessitate autologous hematopoietic stem-cell transplantation [6, 7]. To prevent such hematological toxicities in escalation therapies, pre-therapy dosimetry was introduced using the positron-emitting ^124^I (^124^I-mIBG) to individually estimate tumour absorbed dose and toxicity level [8]. The long ^124^I half-life of 4.2 days and superior quantification performance of positron emission tomography/computed tomography (PET/CT) imaging when compared with single photon emission tomography or planar scintigraphy, facilitates the assessment of organ/tumour uptake and biokinetics [9–11].

Considering the age distribution of typical patients with neural crest tumours, special attention to the radiation exposure from application of radioiodinated mIBG should be paid to children. Neuroblastoma typically occur in children and belong to the most frequent paediatric malignant tumours. In particular, neuroblastoma contribute to 7% of all childhood cancers with 90% of cases being diagnosed by the age of 5 years, and with a reported annual increase of incidences of 1.5% (1978–1997) [12]. The incidence is reported at about 2 per million in the overall population and about 5 to 9 per million in children [13]. Here, the choice of tracer should be taken carefully and aligned with the purpose of investigation. Pheochromocytoma and paraganglioma, however, more frequently occur in adult patients. Incidences are reported between 2 and 8 per million with a peak in age distribution between the 3rd and 5th decades of life and an approximate proportion of pediatric patients of 20% [14]. For ^123^I-mIBG and ^131^I-mIBG, biodistribution and radiation dosimetry studies were performed several decades ago [15–18], whereas for ^124^I-mIBG, no systematic evaluation has been published so far, but one preclinical study and one case series [19, 20]. Therefore, there is a clinical need for a complete description of dosimetry to assess risks of radiation exposure using ^124^I-mIBG.

Since 2005, our institution has been routinely conducting individual ^124^I-mIBG dosimetry prior to ^131^I-mIBG therapy including data from serial PET scans, blood sampling and whole-body counting. These data offer the potential to systematically estimate the absorbed doses to organs and effective doses per unit administered activity among a large group of patients who undergo ^124^I-mIBG PET. This retrospective study is therefore aimed at determining the biodistribution and radiation dosimetry of ^124^I-mIBG in adult patients and extrapolation to paediatric models.

## Materials and methods

### Patients

We retrospectively reviewed our institutional database (Department of Nuclear Medicine, University Hospital Essen, Germany) for ^124^I-mIBG PET examinations in adult patients with neural crest tumours. Inclusion criteria were availability of ^124^I-mIBG PET dosimetry data, one PET dosimetry data set per patient (i.e. patients who underwent more than one PET dosimetry only one dosimetry data set was included to avoid patient bias), availability of complete dosimetry dataset (consisting of at least three serial PET data, five blood sample data and five whole-body counting data points), ^124^I-mIBG PET dosimetry data before any first ^131^I-mIBG therapy, and low tumour burden (to reduce potential impairments of typical biodistribution). A flow chart showing patient inclusion is presented in Additional file 1: Figure S1. The local ethics commission approved the study and waived the need for study specific consent (University of Duisburg-Essen, Medical Faculty, protocol number: 23-11094-BO).

### Production, patient preparation, and administration

The production of ^124^I [21] and the preparation of ^124^I-mIBG [22] was described in literature. ^124^I-mIBG (carrier-added, i.e., a mixture of “hot” and “cold” mIBG) was prepared by isotopic exchange, which is equivalent to the method used to synthesize ^131^I-mIBG [22] and yielded an estimated specific activity of > 150 TBq/mol. Patient preparation followed the recommendations of the EANM procedure guidelines for ^131^I-mIBG therapy to mimic conditions under treatment [23]. More details on ^124^I-mIBG and patient preparation are provided in Additional file 1. ^124^I-mIBG solution of about 40 MBq (0.5–0.6 MBq/kg) was injected intravenously, and patients were asked not to void until the first whole-body retention measurement, yet were strongly encouraged to void before every PET scan.

### PET acquisition and image reconstruction

Patients underwent a series of three (4, 24, and 120 h p.i, n = 7; or 4, 24, and 48 h p.i., n = 2) or four (4, 24, 48, and 120 h p.i., n = 12) ^124^I-mIBG whole-body PET scans on a Biograph Duo PET/CT system and/or a stand-alone EXACT HR^+^ PET system (Siemens Medical Solutions; Illinois, USA). The PET component of both systems was identical, and the sensitivity and axial field of view was 6.7 cps/kBq and 15.5 cm, respectively [24]. Each patient received at least one low-dose CT examination (tube voltage of 110 kVp, tube current time product of 15 mAs, and a pitch of 1.6) performed on the Biograph Duo PET/CT system. In the stand-alone PET, transmission scans (120 s per bed position) were performed using ^68^Ge/^68^ Ga rod sources. For both systems, emission data were acquired in three-dimensional acquisition mode over 300 s per bed position. Standard PET image reconstruction for both systems was performed after Fourier rebinning using an attenuation-weighted ordered-subset expectation maximization algorithm with 2 iterations, 8 subsets, and a 5-mm Gaussian filter. Standard scatter, attenuation (transmission or CT-based), and dead-time corrections provided by the manufacturer were used [25]. PET images had a voxel size of 5.2 × 5.2 × 2.4 mm^3^. The measured reconstructed PET spatial resolution (expressed as full width at half maximum) was 8.2 mm [26]. CT images were reconstructed using a reconstruction interval of 2.4 mm; the reconstructed image had a voxel size of 1.0 × 1.0 × 5.0 mm^3^.

### Blood sampling and whole-body counting

Besides serial PET scans, ^124^I-mIBG dosimetry protocol also included serial blood sampling (to estimate the uptake in the bone marrow) and serial whole-body counting (to estimate the uptake in the remainder of the body). For each patient, heparinized blood samples were collected at 2, 4, 24, 48, and 120 h after ^124^I-mIBG administration and blood ^124^I activity concentrations were measured in a calibrated well counter (Wizard^2^ 2480 3″, PerkinElmer, Waltham, Massachusetts, United States). For each patient, whole-body retention was assessed by measuring the count rates in an uncollimated single-head gamma camera (E.CAM, Siemens, Erlangen, Germany) at same nominal time points as blood sampling except for the early time point (1 h instead of 2 h). The patient was positioned approximately 3 m in front of the uncollimated gamma camera detector. Anterior and posterior counts were acquired for the calculation of the background corrected geometric mean value. Counts were intrinsically corrected for dead time effects.

### Biodistribution and radiation dosimetry

#### Organ activity concentrations

The analyses included organs with visually high uptake in agreement with previous descriptions of physiological mIBG biodistribution [27]: salivary glands, heart, liver, spleen, kidneys, and urinary bladder. Paired organs were considered as one single tissue per patient, that is, parotids and submandibular glands were summarized to a single source of salivary gland tissue and left and right kidney to a single renal source.

To determine the mean imaged activity concentration in organs, volumes-of-interest (VOI) were drawn in the 4-h PET image (showing highest uptake) and propagated to images of later time-points [28]. There are two effects impairing image quantification. First, the imaged activity concentration is affected by the presence of prompt gamma coincidences, that is, in the decay of ^124^I, one positron branch (approximately 50%) is emitted in cascade with a prompt gamma (603 keV) and produces spurious coincidences. This can be corrected for in the sinogram space prior to image reconstruction [29]; however, a sinogram-based approach for these PET systems was not available. Second, the partial-volume effect underestimates the mean imaged activity concentration within tissue boundaries, particularly for small volumes. It has been demonstrated that both effects can be corrected for by applying a heuristic approach [25, 26, 30].

For large organs (liver, spleen, and kidneys), a spherical VOI was drawn centrally in the respective organ [31]. These VOIs (3 cm in diameter for the liver and 2 cm for the kidneys and spleen) were deemed to be small enough—in relation to the size of the organ—to be free of partial-volume effects and to be representative of the respective organ activity concentration. The remaining prompt gamma coincidence effect was corrected by dividing the imaged activity concentration with a factor of 0.8 that was derived from phantom measurements [25].

For small organs (parotid and submandibular salivary glands, urinary bladder, and heart wall), phantom-based recovery coefficients were applied to correct effectively for both effects [25, 30]. For the submandibular and the parotid glands as well as the urinary bladder, a 50% threshold to the maximum activity concentration was used to estimate the volumes and used to determine the effective recovery coefficients. For the heart, four cubical VOIs with 10 mm side length were placed in apico-basal direction along the left ventricular wall and the average value was divided by an experimentally determined effective recovery coefficient of 0.45. A detailed description on heart wall quantification is provided in Additional file 1: text and Figure S2.

#### Uptake curves for organs, blood, and whole-body

The mean activity concentrations of each organ were multiplied with reference organ masses from report 89 of the International Commission on Radiological Protection (ICRP 89) to obtain the individual organ activity assuming homogeneous activity distribution within organs [32]. To estimate the blood activity, the blood activity concentration was multiplied by the overall blood volume [33]. The bone-marrow uptake was estimated using the blood method [34, 35]. The whole-body uptake curve was constructed by normalizing the geometric mean counts to the first data point.

#### Time-integrated activity coefficients

**Fig. 1:**
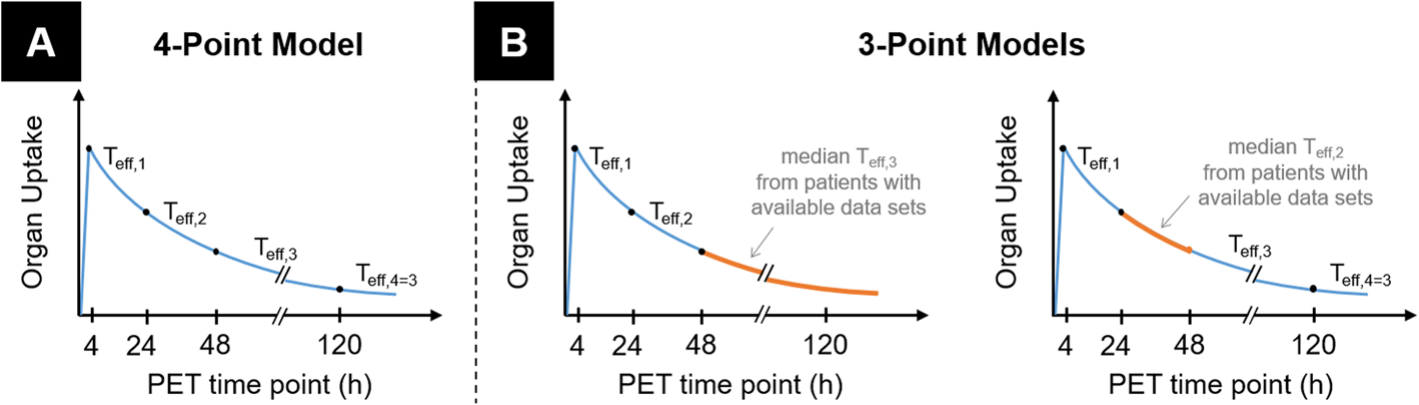
Conceptual representation of kinetic models for organs used for TIAC calculations. In the 4-point model (**A**), TIAC is calculated individually by analytically integrating the mono-exponential fit functions (blue lines) with respective effective half-lives *T*_eff,i_ between subsequent data points, and assuming linear increase until the first data point. In the 3-point models (**B**), missing data points are substituted by median *T*_*eff,i*_ from patients with available data and used for integration

Time-specific effective half-lives for source organs, blood, and whole-body were derived by calculating mono-exponential functions between adjacent time points. Time-integrated activity coefficients (TIACs) were derived by integrating the single mono-exponential functions. For organs, models for calculating the TIACs are illustrated in Fig. 1. A linear increase until the first (4-h) data point is assumed. Effective half-lives were calculated between 4 and 24 h (*T*_eff,1_), 24 and 48 h (*T*_eff,2_), and 48 and 120 h (*T*_eff,3_) and used for integration between the respective intervals. *T*_eff,3_ was also used for the integration after the 120-h data point. For patients with three available PET data points, median *T*_eff,2_ or median *T*_eff,3_ from the patients with available data was used to replace the missing effective half live. Blood and whole-body TIACs were calculated as for organs, but assuming a constant uptake for the time between administration and the first data point. TIAC of the remainder of the body was calculated by subtracting the TIAC contributions of the organs from the whole-body TIAC.

Organ absorbed dose and effective dose coefficients in adults, extrapolation to paediatric patients for ^124^I-mIBG, and projection to ^123^I-mIBG and ^131^I-mIBG.

Absorbed dose coefficients (in mGy/MBq) to normal organs were calculated individually using OLINDA/EXM 2.2 for the reference 73-kg male and 60-kg female [32, 36]. The individual effective dose coefficient (in mSv/MBq) was calculated by applying the tissue-weighting factors from ICRP 103 [37]. The measured biokinetic data from adult patients (mean values of both females and males) were used to extrapolate dosimetry estimates in gender averaged paediatric patients representing a newborn and children at the age of 1, 5, 10, and 15 years (3.5, 10, 19, 32, and 54.5 kg, respectively) using phantom models described in ICRP 89 [32]. For this purpose, a linear scaling of the organ-related uptake was performed to compensate for anatomical differences and faster metabolism of paediatric patients compared to adults [38]. Absorbed dose predictions for ^123^I-mIBG and ^131^I-mIBG were performed individually for adult patients by correcting the measured ^124^I-mIBG uptake values for the different physical half-lives according to the radioactive decay law [28]. The projected uptake values were used to construct the individual uptake curves, from which the projected TIAC values were estimated analogously to.^124^I-mIBG. A detailed description on the extrapolation to paediatric models and projection to other radionuclides is provided in Additional file 1

## Results

### Patients

We identified a total of 81 ^124^I-mIBG PET examinations that were conducted at our institution from 2005 to 2015. 21 patients (11 females, 10 males) fulfilled the inclusion criteria and were included in this retrospective analysis. Demographic data are summarized in Table 1. The mean ± standard deviation (SD) administered ^124^I-mIBG activity was 43 ± 4 MBq. For twelve patients, four PET imaging data points, for two patients the 4-h, 24-h, and 48-h, and for seven patients the 4-h, 24-h, and 120-h data points were available.

**Table 1:** Patient demographics of 21 individuals

Patient demographics [Reference height and weight defined in ICRP89]		
Parameter	Data	
Gender	Male	Female
Patients	10 (47.6%)	11 (52.4%)
Mean age ± SD (a)	53 ± 18	41 ± 18
Mean height ± SD (cm)	173 ± 8 [176]	166 ± 3 [163]
Mean weight ± SD (kg)	73 ± 15 [73]	61 ± 7 [60]
*Malignancies*		
Pheochromocytoma	9 (42.9%)	
Neuroblastoma	4 (19.0%)	
Paraganglioma	1 (4.8%)	
Gastroenteropancreatic NET	7 (33.3%)	

### Biodistribution

**Fig. 2:**
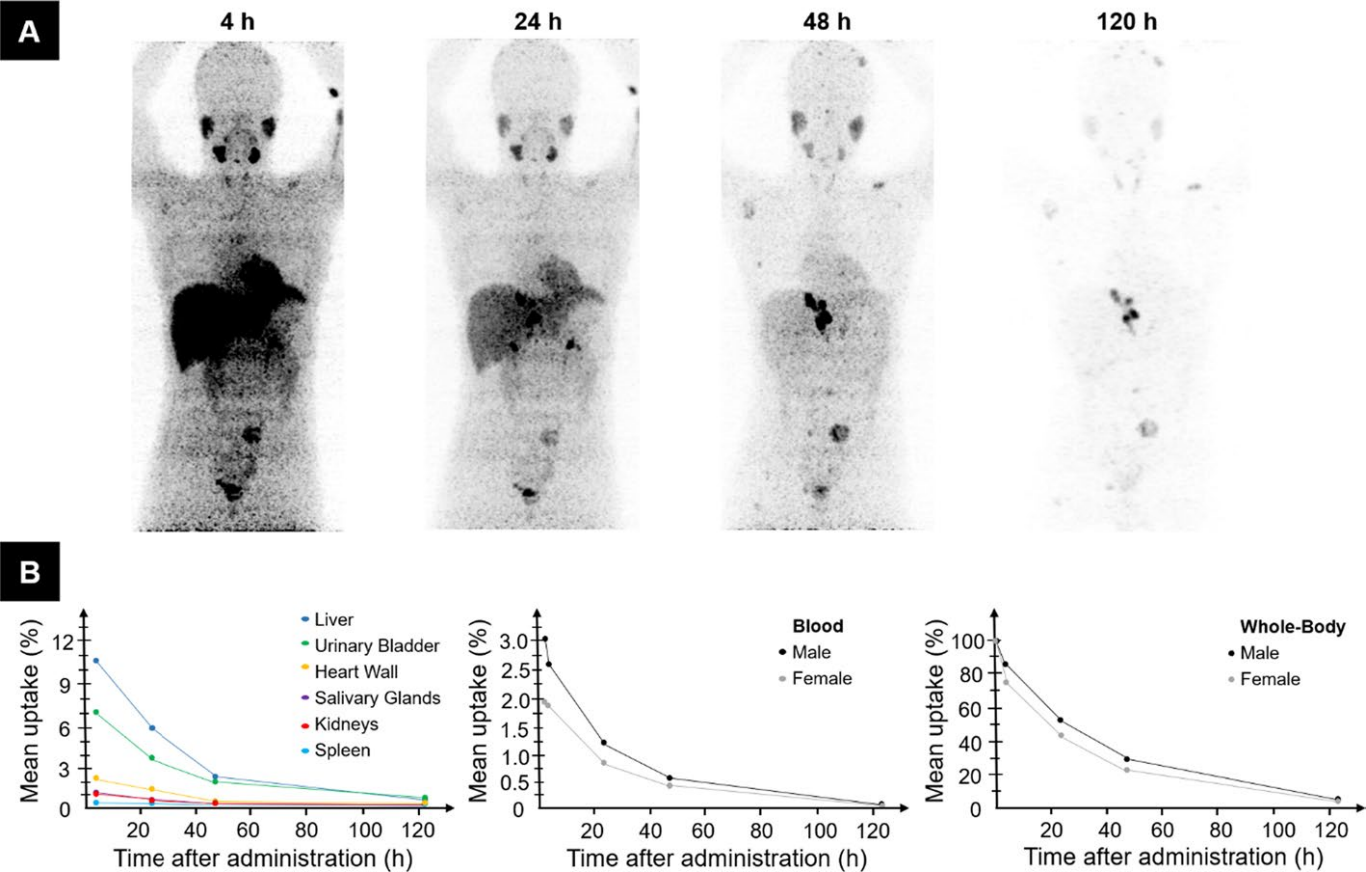
Sequential MIPs from PET imaging of a representative individual with suspected pheochromocytoma after administration of ^124^I-mIBG (**A**). On the bottom row (**B**), uptake curves are illustrated for different organs (left), averaged over 21 individuals, and blood (middle) and whole-body retention curves (right), separated by gender

Maximum-intensity projections from sequential ^124^I-mIBG PET imaging of a representative patient and percentage injected activity curves are presented in Fig. 2. Actual mean ± SD PET imaging time points after administration were 4.2 ± 0.7, 25 ± 1, 47 ± 1, and 125 ± 14 h. Blood sampling and whole-body counting were performed at 1.8 ± 0.3, 3.9 ± 0.6, 24 ± 2, 46 ± 2, and 124 ± 14 h. The organ with the highest initial percentage uptake was the liver with a mean ± SD of 10.7 ± 3.7% for male and 10.1 ± 2.4% for female patients, followed by the urinary bladder (7.1 ± 2.6% for male and 8.0 ± 2.0% for female patients), and the heart wall (2.3 ± 1.2% for male and 3.1 ± 0.7% for female patients). For the salivary glands, kidneys, and the spleen the mean initial uptake was below 1.1%. Mean blood uptake at 2 h post injection was 3.0 ± 1.1% for male and 1.9 ± 0.8% for female patients and decreased to 0.16 ± 0.12% (0.08 ± 0.05%) for male (female) patients at the 120-h time point. On average, whole-body uptake was below 50% at the 24-h time point for both male and female patients and decreased to 6.6 ± 3.5% (4.5 ± 2.1%) for male (female) patients at the 120-h time point.

Table 2 lists the median (1. and 3. quartile) organ-specific effective half-lives between subsequent time points using the data of both male and female patients. No substantial gender-related differences in effective half-lives were observed. For the urinary bladder, liver, kidneys, blood, and the heart wall the relative increase from median *T*_eff,1_ to *T*_eff,3_ was 3.3-, 2.5-, 2.1-, 1.8-, and 1.7-fold, respectively, indicating bi-exponential clearance in these organs. For one individual, T_eff,3_ of the heart was negative, whereupon physical decay was assumed after the last imaging time point for radiation dosimetry.

**Table 2:** Median [1. and 3. quartile] organ- and time-specific effective half-lives (h) of ^124^I-mIBG in 21 adult patients

Organ	Effective half-life (h)			
	*T*_eff, 2 h–4 h_	*T*_eff1, 4 h–24 h_	*T*_eff2, 24 h–48 h_	*T*_eff3, 48–120 h_
Salivary glands	NA	19.6 [16.3, 43.0]	23.9 [21.7, 28.3]	24.9 [23.0, 28.3]
Heart wall	NA	16.7 [14.5, 19.2]	20.0 [12.0, 36.0]	28.4 [20.9, 37.5]
Liver	NA	16.9 [13.1, 28.9]	20.4 [14.0, 23.4]	42.8 [30.2, 49.6]
Spleen	NA	31.3 [21.1, 54.2]	25.1 [16.5, 31.6]	34.7 [28.5, 38.7]
Kidneys	NA	17.4 [15.6, 28.0]	26.3 [19.7, 31.9]	36.5 [25.5, 40,6]
Urinary bladder	NA	17.4 [10.7, 29.1]	12.4 [7.7, 20.7]	57.6 [49.2, 72.0]
Blood	7.7 [4.6, 14.2]	16.2 [9.8, 25.6]	27.1 [20.7, 32.0]	29.2 [27.5, 36.3]
Whole-body	9.4 [7.2, 17.9]	23.8 [21.0, 33.3]	25.6 [24.0, 27.3]	30.1 [27.6, 31.0]

### Radiation dosimetry

Mean TIAC values for male and female patients as well as the extrapolated values for paediatric models are shown in Table 3. The remainder-of-the-body TIAC amounted 75% of the respective whole-body TIAC. Of note, the remainder-of-the-body TIAC was larger for male (30.3 h) than for female (25.0 h). Among the organs, highest TIAC values were observed in the liver, the urinary bladder wall, and the heart wall; the respective ratio of organ to whole-body TIAC for adult male (female) patients were 11% (12%), 9% (8%), and 2% (3%).

**Table 3:** Mean (± SD) organ-specific time-integrated activity coefficients (TIAC) for ^124^I-mIBG in adult patients and their extrapolation to reference paediatric patients defined in ICRP89

Organ	TIAC (h)						
	Adult Male	Adult Female	15-yrs-old	10-yrs-old	5-yrs-old	1-yr-old	Newborn
Salivary glands	0.41 (0.15)	0.42 (0.17)	0.44 (0.17)	0.49 (0.19)	0.64 (0.25)	0.86 (0.34)	0.62 (0.24)
Heart wall	0.86 (0.42)	0.93 (0.22)	0.85 (0.30)	0.90 (0.32)	0.92 (0.32)	1.03 (0.36)	1.18 (0.41)
Liver	4.45 (1.52)	4.12 (1.69)	4.26 (1.60)	4.63 (1.74)	5.36 (2.01)	5.89 (2.21)	6.63 (2.49)
Spleen	0.19 (0.04)	0.17 (0.04)	0.20 (0.05)	0.21 (0.05)	0.22 (0.05)	0.23 (0.06)	0.23 (0.06)
Kidneys	0.41 (0.14)	0.37 (0.11)	0.40 (0.13)	0.50 (0.16)	0.51 (0.17)	0.62 (0.20)	0.63 (0.20)
Urinary bladder	3.10 (0.90)	2.63 (0.93)	3.23 (0.97)	3.54 (1.06)	3.75 (1.13)	3.62 (1.09)	3.90 (1.17)
Bone marrow	0.25 (0.09)	0.14 (0.04)	0.24 (0.08)	0.25 (0.08)	0.23 (0.08)	0.19 (0.06)	0.18 (0.06)
Remainder of body	30.31 (5.71)	25.00 (8.46)	27.47 (7.17)	26.56 (7.40)	25.46 (7.73)	24.63 (8.90)	23.72 (9.25)
Whole body	40.40 (5.93)	33.78 (9.11)	37.09	37.09	37.09	37.09	37.09

The organ absorbed dose coefficients are listed in Table 4. For all age groups, the organs exposed to the highest absorbed dose per unit administered activity were the urinary bladder, the salivary glands, the liver, and the heart wall. The mean effective dose for ^124^I-mIBG was 0.25 ± 0.04 mSv/MBq, almost equal between male and female patients (0.24 ± 0.03 for male and 0.26 ± 0.06 mSv/MBq for female patients) and increased gradually to 0.29, 0.44, 0.69, 1.21, and 2.94 mSv/MBq for the 15-, 10-, 5-, 1-years-old, and newborn reference model, respectively (Table 5).

**Table 4:** Measured mean (± SD) organ absorbed dose coefficients (mGy/MBq) for ^124^I-mIBG in adult patients and extrapolated values for reference paediatric patients pefined in ICRP89

Organ	Organ absorbed dose (mGy/MBq)						
	Adult Male	Adult Female	15-yrs-old	10-yrs-old	5-yrs-old	1-yr-old	Newborn
Adrenals	0.297 (0.053)	0.314 (0.075)	0.361	0.560	0.883	1.64	3.44
Brain	0.134 (0.026)	0.137 (0.045)	0.155	0.248	0.392	0.684	1.57
Breast		0.141 (0.042)	0.148				
Esophagus	0.202 (0.033)	0.218 (0.050)	0.251	0.400	0.651	1.24	3.03
Eyes	0.132 (0.025)	0.137 (0.045)	0.154	0.245	0.404	0.705	1.61
Gallbladder wall	0.329 (0.064)	0.259 (0.065)	0.328	0.496	0.770	1.39	3.39
Left colon	0.201 (0.032)	0.263 (0.133)	0.226	0.361	0.570	0.974	2.37
Right colon	0.220 (0.033)	0.210 (0.059)	0.243	0.375	0.581	0.998	2.39
Small intestine	0.214 (0.032)	0.202 (0.054)	0.249	0.386	0.593	1.04	2.37
Stomach wall	0.210 (0.032)	0.209 (0.055)	0.239	0.352	0.560	0.960	2.31
Rectum	0.254 (0.029)	0.320 (0.080)	0.351	0.545	0.831	1.33	3.12
Heart wall	0.513 (0.200)	0.649 (0.105)	0.677	1.26	1.75	2.59	8.27
Kidneys	0.347 (0.088)	0.355 (0.080)	0.412	0.649	1.04	1.85	4.49
Liver	0.608 (0.179)	0.689 (0.260)	0.755	1.19	1.89	3.34	8.27
Lungs	0.186 (0.029)	0.200 (0.052)	0.224	0.336	0.525	0.942	2.26
Ovaries/Prostate	0.289 (0.036)	0.240 (0.064)	0.311	0.633	0.749	1.23	2.71
Pancreas	0.230 (0.034)	0.264 (0.063)	0.285	0.434	0.676	1.21	2.83
Salivary glands	0.704 (0.237)	0.829 (0.325)	0.918	1.48	2.42	4.41	10.8
Red marrow	0.175 (0.025)	0.174 (0.044)	0.195	0.262	0.372	0.654	1.24
Osteogenic cells	0.175 (0.028)	0.164 (0.047)	0.194	0.254	0.339	0.569	0.809
Spleen	0.303 (0.055)	0.316 (0.063)	0.365	0.571	0.914	1.63	3.93
Thymus	0.193 (0.035)	0.201 (0.053)	0.229	0.335	0.534	0.935	2.22
Thyroid	0.161 (0.029)	0.154 (0.048)	0.190	0.296	0.487	0.878	2.02
Urinary bladder wall	1.67 (0.398)	1.41 (0.396)	1.83	2.96	4.76	8.38	21.9
Uterus/ Testes	0.170 (0.025)	0.322 (0.080)	0.318	0.542	0.785	1.26	2.82
Total body	0.175 (0.027)	0.188 (0.050)	0.203	0.316	0.494	0.882	2.14

**Table 5:** Measured mean (± SD) effective dose coefficient (mSv/MBq) for ^124^I-mIBG in adult patients, extrapolated mean values for reference paediatric patients, and projected mean (± SD) values for ^123^I-mIBG and ^131^I-mIBG in adult patients. Values within parentheses are provided by ICRP publication 53 for ^124^I-mIBG and publication 80 for ^123^I-mIBG and ^131^I-mIBG [15, 18]

Effective dose coefficient (mSv/MBq)					
Adult patients					
^124^I-mIBG		^123^I-mIBG - projected		^131^I-mIBG - projected	
Male	Female	Male	Female	Male	Female
0.24 ± 0.03	0.26 ± 0.06	0.014 ± 0.002	0.015 ± 0.002	0.18 ± 0.04	0.19 ± 0.05
(0.24)		(0.013)		(0.14)	

Paediatric reference patients				
^124^I-mIBG - extrapolated				
15-yrs old	10-yrs old	5-yrs old	1-yr old	Newborn
0.29 (0.30)	0.44 (0.44)	0.69 (0.67)	1.21 (1.2)	2.94 (–)
0.005*	0.014*	0.036*	0.12*	0.84*

*Effective dose coefficient per kg of patient weight (mSv/MBq/kg)

Projected values for TIAC and organ absorbed dose coefficients for ^123^I-mIBG and ^131^I-mIBG for adults are listed in Additional file 1: Tables S1 and S2. Projected effective dose coefficients for ^123^I-mIBG were 0.014 ± 0.002 and 0.015 ± 0.002 mSv/MBq for male and female, respectively. Projected effective dose coefficients for ^131^I-mIBG were 0.18 ± 0.04 mSv/MBq (male) and 0.19 ± 0.05 mSv/MBq (female).

## Discussion

To the best of our knowledge, this is the first systematic analysis to report the biodistribution and radiation dosimetry of ^124^I-mIBG in a large patient group. For adult patients, the measured effective dose coefficient was 0.25 ± 0.04 mSv/MBq (mean value of both females and males) and the extrapolated values for children at the age of 15, 10, 5, 1 year(s), and newborn were 0.29, 0.44, 0.69, 1.21 and 2.94 mSv/MBq, respectively (Table 5). From radiation safety viewpoint, these results support its applicability for diagnostics and pretherapeutic dosimetry.

In line with published literature on radioiodinated mIBG [16, 17], ^124^I-mIBG was rapidly cleared from the blood, and most of the organs indicated a bi-exponential clearance behavior (Table 2). The organ with the highest absorbed dose coefficient was the urinary bladder, followed by the salivary glands, the liver, and the heart wall (Table 4). These organs were also amongst the organs with highest absorbed doses in previous descriptions for ^123^I-mIBG and ^131^I-mIBG [18, 39]. The salivary glands showed the highest inter-patient variation (SD =  ± 37%), which is in line with previous observations on projected salivary glands’ self-absorbed doses of ^131^I-mIBG (0.53 Gy/GBq ± 45%) [40]. The projected salivary glands’ absorbed doses in our study were slightly higher (Additional file 1: Table S2) due to differences in organ volume segmentation and a minor contribution arising from cross-radiation from other source organs which was not considered in the previous publication.

**Fig. 3:**
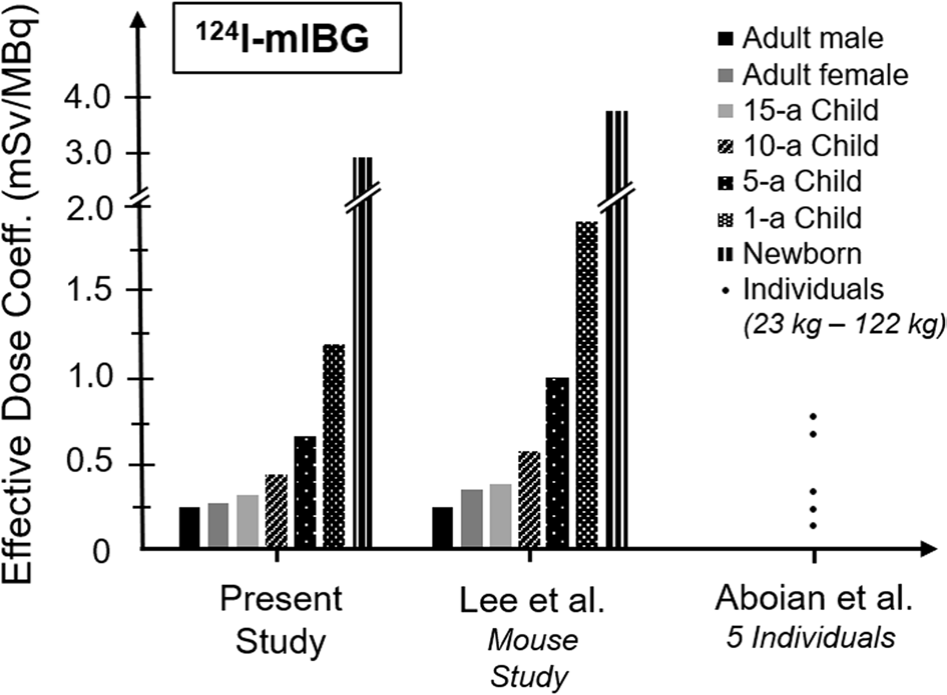
Comparison of effective dose coefficients for ^124^I-mIBG obtained in the present study (n = 21), extrapolations from a murine model [19], and individual estimations [20]

Table 5 shows the effective dose coefficients obtained in our study and the values listed in ICRP publications 53 and 80 [15, 18]. Although absorbed organ doses were not provided, the projected effective dose coefficients for ^124^I-mIBG (which was presented as a radionuclide impurity of ^123^I-mIBG) in ICRP53 are in excellent agreement with the values obtained in this study. Of note, in ICRP80—an addendum to ICRP53—effective dose coefficients for ^123^I-mIBG and ^131^I-mIBG were revised, but not the projected values for ^124^I-mIBG. We here provide a PET-based update of the effective dose coefficients for ^124^I-mIBG, and in addition, provide organ absorbed dose coefficients. Figure 3 illustrates the agreement of our effective dose coefficients with extrapolated preclinical data [19] and estimations on five individuals [20]. More precisely, Lee et al*.* [19] performed a preclinical study in mice and extrapolated the results onto paediatric and adult patients. For adult male patients, they reported an effective dose coefficient of 0.25 mSv/MBq. However, when extrapolating to adult female patients by mass-scaling they claimed an increase of 36% (0.38 mSv/MBq) that was not confirmed in our study. Our patient data reveal that TIAC of the remainder of the body was smaller for female patients (25.0 vs. 30.3 h). Since the remainder of the body contributes to around 75% to the whole-body TIAC (Table 3), this finally leads to similar effective dose coefficients between males and females (0.24 vs 0.26 mSv/MBq). Overall, the differences between this and the preclinical study are rather small, even for paediatric models. In a case series investigating patients with neuroblastoma, Aboian et al*.* [20] estimated the effective dose coefficient for a 122-kg female, 63-kg male, 40-kg male, 29-kg female, and 23-kg female to be 0.161, 0.252, 0.339, 0.706, and 0.795 mSv/MBq, respectively. When performing a linear weight-based approximation, these values are comparable with the data from the present study. For ^123^I-mIBG and ^131^I-mIBG the ICRP reported effective dose coefficients of 0.013 mSv/MBq and 0.14 mSv/MBq, respectively [15, 18]. These values also agree well with our projected effective dose coefficients (Table 5).

Generally, implementation of ^124^I-mIBG PET/CT examinations has several advantages over ^123^I/^131^I-mIBG-planar or -SPECT/CT imaging including higher image quality, improved image quantification, and lesion detectability [3, 9, 10, 20, 41–43]. In our study, adult patients received a mean activity of 40 MBq (0.5–0.6 MBq/kg) that limit the effective dose to a reasonable amount of 10 mSv. In paediatric patients, the administered activity should be adjusted accordingly for weight (Table 5). For example, the application of 10 MBq ^124^I-mIBG in a 5-year-old patient (weighting 19 kg) would result in an effective dose of 7 mSv. It is expected that digital PET/CT systems (providing higher sensitivity) may allow to further reduce the injected activities while maintaining a high quantitative performance, which is particularly relevant in pretherapeutic lesion dosimetry in the context of radiotherapy planning [44]. In comparison to ^124^I-mIBG, administration of typical 350 MBq ^123^I-mIBG results in an effective dose of 4.5 mSv [45] for an adult patient, and approximately 3.7 mSv from the EANM-recommended administration of 5.2 MBq/kg ^123^I-mIBG [18, 45] for a 5-year-old (19 kg). In comparison to other molecular targets, that are applied in patients with neural crest tumours, effective doses for ^124^I-mIBG are higher by a factor of about two. For example, effective doses for the SSTR-agonists ^68^Ga-DOTATOC/DOTATATE are in the range of 4–5 mSv and about 7 mSv for ^18^F-FDG for SNMMI/EANM recommended injected activities [46]. Against this background, the benefits of ^124^I-mIBG PET justify the theranostic use of ^124^I-mIBG, especially in the context of individualized planning of ^131^I-mIBG therapies, where significantly higher organ absorbed doses are achieved.

There are several limitations. First, the study was limited by its retrospective design. Second, only patients with low tumour burden were included, which is important to establish reference absorbed dose values but may hamper the translation to individuals with high tumour load. Third, although extrapolated paediatric dose coefficients were estimated, a representative paediatric group is still missing. In the present study, whole-body TIAC was assumed to be constant during extrapolation. It is however likely that overall excretion kinetics in children are faster compared to adults, possibly leading to an overestimation of the absorbed dose coefficients for paediatric reference patients in our study [15]. Fourth, we applied a heuristic method to correct for prompt gamma coincidence and partial volume effects. However, the correction factors are expected to be dependent on the organ geometry [26]. A systematic examination of this effect was not possible because of the retrospective study design (unavailability of the PET systems). Lastly, the method of preparation of ^124^I-mIBG yielded a notable amount of unlabeled mIBG. Indeed, the ratio of radioactive to non-radioactive mIBG is only about one in several thousand. To the authors’ opinion, the influence of the specific activity on organ uptake may be neglected in diagnostic imaging as the administered activity of 40 MBq equals only 35 pmol ^124^I-mIBG.

## Conclusion

This was the first systematic analysis in a clinical patient cohort to determine the biodistribution and radiation dosimetry of ^124^I-mIBG in patients with neuronal crest tumours. Biodistribution and radiation dosimetry is favourable for PET/CT imaging, especially for pretherapeutic dosimetry prior to treatment. The effective dose coefficients presented here showed a high agreement with historical projected data from ICRP publication 53, and can be used to establish reference activity administration protocols for adult and paediatric patients.

### Supplementary Information

**Additional file 1**. **Supplemental table 1.** Projected mean (±SD) organ-specific time-integrated activity coefficients (h) for ^123^I-mIBG and ^131^I-mIBG. **Supplemental table 2.** Projected mean (±SD) organ absorbed doses (mSv/MBq) for adult patients for ^123^I-mIBG and ^131^I-mIBG. **Supplemental figure 1.** Flow chart showing who ^124^I-mIBG PET examinations in adult patients with neural crest tumours that were conducted at our institution (University Hospital Essen) between 2005 and 2015 and patients who were evaluated for radiation dosimetry according to the inclusion criteria. TX: therapy, blood data: blood sampling data, whole-body data: whole-body counting. **Supplemental figure 2.** PTW Heart phantom “C” (upper left) was mounted in a water-filled PTW Head phantom “B” (upper right). The PET images with four cubical VOIs of 10 mm side length are illustrated for the phantom (lower left) used to determine the heart wall recovery coefficient and a representative patient example (lower right). Phantom images were taken from the publication Tylski et al. (Tissue dose estimation after extravasation of 177Lu-DOTATATE, EJNMMI Physics 2021; 8:33).

## Data Availability

The datasets generated and analyzed in this study are not publicly available due to it containing patient identifiable information. Requests to access these datasets should be directed at: alexandros.moraitis@uk-essen.de.
