# Supplementary material for: Biodistribution and radiation dosimetry of 124I-mIBG in adult patients with neural crest tumours and extrapolation to paediatric models
Source: EJNMMI Phys. 2024 Jan 3;11:3. doi: 10.1186/s40658-023-00604-0 (PMC10761661; doi:10.1186/s40658-023-00604-0)
Supplement: Supplementary file 1 — Additional file 1. Supplemental table 1. Projected mean (±SD) organ-specific time-integrated activity coefficients (h) for 123I-mIBG and 131I-mIBG. Supplemental table 2. Projected mean (±SD) organ absorbed doses (mSv/MBq) for adult patients for 123I-mIBG and 131I-mIBG. Supplemental figure 1. Flow chart showing who 124I-mIBG PET examinations in adult patients with neural crest tumours that were conducted at our institution (University Hospital Essen) between 2005 and 2015 and patients who were evaluated for radiation dosimetry according to the inclusion criteria. TX: therapy, blood data: blood sampling data, whole-body data: whole-body counting. Supplemental figure 2. PTW Heart phantom “C” (upper left) was mounted in a water-filled PTW Head phantom “B” (upper right). The PET images with four cubical VOIs of 10 mm side length are illustrated for the phantom (lower left) used to determine the heart wall recovery coefficient and a representative patient example (lower right). Phantom images were taken from the publication Tylski et al. (Tissue dose estimation after extravasation of 177Lu-DOTATATE, EJNMMI Physics 2021; 8:33). [file 40658_2023_604_MOESM1_ESM.docx]

**Biodistribution and radiation dosimetry of ^124^I-mIBG in adult patients with neuroendocrine tumours and extrapolation to paediatric models**

Alexandros Moraitis^1,2^, Walter Jentzen^1,2^, Gloria Reiter^1,2^, Jochen Schmitz^1,2^, Thorsten Dirk Pöppel^1,2^, Manuel Weber^1,2^, Ken Herrmann^1,2^, Wolfgang Peter Fendler^1,2^, Pedro Fragoso Costa^1,2^, Andreas Bockisch^1,2^, David Kersting^1,2^

^1^Department of Nuclear Medicine, West German Cancer Center (WTZ), University Hospital Essen, University of Duisburg-Essen, Essen,
Germany

^2^German Cancer Consortium (DKTK), Partner Site University Hospital Essen, Essen, Germany

**Short title:**

Radiation dosimetry of ^124^I-mIBG

**Keywords:**

Dosimetry, ^124^I-mIBG, PET, Effective dose, Neural crest tumour

**Corresponding author:**

Alexandros Moraitis, M. Sc.

Department of Nuclear Medicine, University Hospital Essen, University of Duisburg-Essen

Hufelandstrasse 55, D-45147 Essen, Germany

Fax: +49-201-723-5535, Phone: +49-201-723-84057

Email: [alexandros.moraitis@uk-essen.de](mailto:alexandros.moraitis@uk-essen.de)

# **Supplemental material**

# **Material and methods**

## **Preparation of ^124^I-mIBG and patient preparation**

The production of ^124^I (21) and the preparation of ^124^I-mIBG (22) was described in literature. ^124^I-mIBG (carrier-added, i.e. a mixture of “hot” and “cold” mIBG) was prepared by isotopic exchange equivalently to the method used to synthesize ^131^I-mIBG (22). The mIBG precursor was obtained from Sigma-Aldrich (Steinheim, Germany). Purification of the ^124^I-mIBG was carried out by semi-preparative high-performance liquid chromatography resulting in a radiochemical purity greater than 99%. The specific activity was estimated to be >150 TBq/mol. At the time of administration, the free ^124^I-iodide content was negligible (<1%). ^124^I-mIBG activity measurement was equivalent to that of ^124^I-NaI, which has been published elsewhere (*26*).

Patient preparation followed the recommendations of the EANM procedure guidelines for ^131^I-mIBG therapy to mimic conditions under treatment (*23*). To avoid thyroidal uptake of free ^124^I, 6 drops of Lugol’s solution containing 1 mg iodide were administered orally 20 min before administration of ^124^I-mIBG. Patients were encouraged to stay hydrated to reduce radiation burden to the urinary bladder.

## **Biodistribution and radiation dosimetry**

### Organ activity concentration – Heart wall

Partial volume effect in the heart wall was corrected using a heart phantom. The heart phantom is illustrated in supplemental figure 2 (left and middle) and was used to determine the heart-wall recovery coefficient. The heart phantom-insert (PTW Heart phantom “C”) consisted of two non-axial cylinders, an inner and an outer cylinder. It was mounted in a water-filled cylinder phantom (PTW Head phantom “B”). Both the inner cylinder (inner diameter of 4.4 cm, length of 10 cm; inner volume of 156 mL) and the outer cylinder (inner diameter of 8.0 cm, length of 10 cm; inner volume of 310 mL) were filled with ^124^I-NaI solution. The heart phantom was positioned with its cylinder axis parallel to the z-axis of the PET scanner. At the time point of PET measurement, the prepared ^124^I activity concentration in the inner and out cylinder was 4.90 kBq/mL and 58.93 kBq/mL, respectively. Image acquisition and image reconstruction were identical with the clinical settings. Four cubical VOIs with 10 mm side length (supplemental figure 2, right) were placed in a region representing the phantom’s heart wall (thickness of about 10 mm), that is, the smallest distance between the inner and outer cylinder surfaces. The imaged average activity concentration was divided by the prepared activity concentration. The resulting heart-wall recovery coefficient was 0.45 and used to correct the imaged activity concentration in the patient study. Clinically, analogously to the phantom measurement, four cubical VOIs with 10 mm side length were placed in apico-basal direction along the left ventricular wall (supplemental Figure 2) and the mean averaged activity concentration was divided by 0.45.

### Uptake curves for organs, blood and whole-body

Uptake curves were generated for all patients, separately for different organs, blood, and whole-body retention. The mean activity concentrations of each organ were multiplied with the reference organ masses to obtain the individual organ activity. The reference organ masses were taken from publication 89 of the International Commission on Radiological Protection (ICRP 89) for the reference adult male and female assuming homogeneous activity distribution within the organs (*29*). A constant bladder content of 211 ml for male patients (160 ml for female patients) was used (ICRP89). To estimate the blood activity, the blood activity concentration was multiplied by the overall blood volume using the empiric formulae suggested by the International Council for Standardization in Haematology – a formulae that considers age, height, and weight of the patient (*30*). Organ/blood activities were normalized to the administered activity to obtain organ/blood uptake values. The bone-marrow uptake was estimated using the blood method (*31*), that is, activity concentration in the bone marrow was equal to that in blood, assuming that there was no specific ^124^I-mIBG binding in the bone marrow (*32*). The whole-body uptake curve was constructed by normalizing the geometric mean counts to the first data point.

### Organ absorbed dose and effective dose coefficients in adults, extrapolation to paediatric patients for ^124^I-mIBG, and projection to ^123^I-mIBG and ^131^I-mIBG

Based on the individual TIAC values, absorbed dose coefficients (mGy/MBq) to normal organs were calculated individually using OLINDA/EXM 2.2 for the reference 73-kg male and 60-kg female (*29*). The individual effective dose coefficient (in mSv/MBq) was calculated by applying the tissue-weighting factors from ICRP 103 (*33*).

The measured biokinetic data from adult patients (mean values of both females and males) were used to extrapolate dosimetry estimates in reference paediatric patients representing a newborn and children at the age of 1, 5, 10, and 15 years using phantom models according to ICRP 89. For this purpose, a linear scaling of the organ-related uptake is necessary to compensate for anatomical differences of paediatric patients compared to adults (*34*). The mean uptake of organ in adults was modified using following equation:

where *U* refers to the uptake in an organ at time *t* and *m* to the mass of either an organ or the whole-body mass. Further, the model assumes that the total number of disintegrations per unit administered activity is preserved during extrapolation. TIAC of the remainder of the body was calculated by subtracting the extrapolated TIAC values of the organs from the whole-body TIAC. Dosimetry calculations were executed in OLINDA/EXM 2.2.

Absorbed dose predictions for ^123^I-mIBG and ^131^I-mIBG were performed individually for adult patients by correcting the measured ^124^I-mIBG uptake values, that is, the difference in the physical half-lives between ^123^I (13.22 h) or ^131^I (8.02 d) and ^124^I (4.17 d) must be taken into account according to the radioactive decay law (*26*).

It is *T*_124_ the physical half-life of ^124^I, *T*_isotope_ the physical half-life of ^123^I (or ^131^I). *U*_124_(*t*) denotes measured uptake value and *U*_isotope_ (*t*) the projected uptake values at each time point *t*. The projected uptake values were used to construct the individual uptake curves, from which the projected TIAC values were estimated analogously to ^124^I-mIBG. Dosimetry calculations were executed in OLINDA/EXM 2.2.

## **Software and statistics**

The image analyses were conducted using PMOD 4.2 software (PMOD Technologies Ltd., Zurich, Switzerland). Statistical analysis was performed using GraphPad Prism 5.03 (Graphpad Software Inc., CA, USA). The descriptive statistics included the mean, median and the measure of dispersions were expressed in standard deviation (SD) and quartiles (Q1 and Q3). Mann-Whitney-U test was used and significance was assumed for p < 0.05.

# **Supplemental tables**

## **Supplemental table 1**

**Supplemental table 1**. Projected mean (±SD) organ-specific time-integrated activity coefficients (h) for ^123^I-mIBG and ^131^I-mIBG.

|  | TIAC (h) | | | |
| --- | --- | --- | --- | --- |
|  | ^123^I-mIBG | | ^131^I-mIBG | |
| Organ | Adult  Male | Adult  Female | Adult  Male | Adult  Female |
| Salivary Glands | 0.13 (0.03) | 0.14 (0.05) | 0.49 (0.20) | 0.49 (0.20) |
| Heart Wall | 0.28 (0.14) | 0.35 (0.09) | 1.07 (0.53) | 1.09 (0.25) |
| Liver | 1.40 (0.43) | 1.18 (0.19) | 5.65 (2.27) | 5.72 (2.46) |
| Spleen | 0.06 (0.01) | 0.05 (0.01) | 0.22 (0.06) | 0.21 (0.06) |
| Kidneys | 0.13 (0.05) | 0.11 (0.01) | 0.49 (0.18) | 0.48 (0.22) |
| Urinary  Bladder | 0.89 (0.22) | 0.80 (0.29) | 4.11 (1.67) | 3.24 (1.07) |
| Bone Marrow | 0.10 (0.03) | 0.05 (0.01) | 0.28 (0.10) | 0.16 (0.06) |
| Remainder of Body | 10.40 (2.18) | 8.62 (1.58) | 32.53 (6.79) | 28.25 (11.58) |
| Whole Body | 13.37 (2.22) | 11.29 (1.88) | 45.84 (6.42) | 39.65 (12.48) |

## **Supplemental table 2**

**Supplemental table 2**. Projected mean (±SD) organ absorbed doses (mSv/MBq) for adult patients for ^123^I-mIBG and ^131^I-mIBG.

|  | Organ absorbed dose (mGy/MBq) | | | |
| --- | --- | --- | --- | --- |
|  | ^123^I-mIBG | | ^131^I-mIBG | |
| Organ | Adult  Male | Adult  Female | Adult  Male | Adult  Female |
| Adrenals | 0.018 (0.003) | 0.018 (0.002) | 0.154 (0.026) | 0.171 (0.054) |
| Brain | 0.009 (0.002) | 0.009 (0.002) | 0.084 (0.017) | 0.089 (0.036) |
| Breast |  | 0.008 (0.001) |  | 0.090 (0.034) |
| Esophagus | 0.013 (0.002) | 0.013 (0.002) | 0.115 (0.020) | 0.127 (0.038) |
| Eyes | 0.009 (0.002) | 0.009 (0.002) | 0.082 (0.017) | 0.088 (0.036) |
| Gallbladder wall | 0.018 (0.003) | 0.016 (0.002) | 0.171 (0.033) | 0.153 (0.047) |
| Left colon | 0.013 (0.002) | 0.013 (0.002) | 0.112 0.019) | 0.126 (0.042) |
| Right colon | 0.013 (0.002) | 0.012 (0.002) | 0.123 (0.019) | 0.123 (0.044) |
| Small intestine | 0.012 (0.002) | 0.012 (0.002) | 0.120 (0.019) | 0.120 (0.041) |
| Stomach wall | 0.012 (0.002) | 0.012 (0.002) | 0.117 (0.020) | 0.122 (0.042) |
| Rectum | 0.015 (0.003) | 0.020 (0.004) | 0.144 (0.019) | 0.171 (0.046) |
| Heart wall | 0.035 (0.010) | 0.041 (0.008) | 0.454 (0.204) | 0.585 (0.109) |
| Kidneys | 0.020 (0.005) | 0.020 (0.002) | 0.261 (0.080) | 0.289 (0.103) |
| Liver | 0.035 (0.009) | 0.037 (0.006) | 0.496 (0.185) | 0.628 (0.364) |
| Lungs | 0.012 (0.002) | 0.013 (0.002) | 0.107 (0.018) | 0.119 (0.040) |
| Ovaries/  Prostate | 0.016 (0.003) | 0.014 (0.003) | 0.164 (0.025) | 0.136 (0.044) |
| Pancreas | 0.014 (0.002) | 0.016 (0.003) | 0.127 (0.021) | 0.151 (0.047) |
| Salivary glands | 0.041 (0.009) | 0.050 (0.017) | 0.715 (0.288) | 0.853 (0.350) |
| Red marrow | 0.010 (0.002) | 0.010 (0.001) | 0.105 (0.017) | 0.104 (0.031) |
| Osteogenic cells | 0.020 (0.003) | 0.019 (0.003) | 0.113 (0.020) | 0.102 (0.035) |
| Spleen | 0.017 (0.002) | 0.017 (0.002) | 0.233 (0.051) | 0.259 (0.070) |
| Thymus | 0.011 (0.002) | 0.013 (0.002) | 0.109 (0.021) | 0.117 (0.039) |
| Thyroid | 0.010 (0.002) | 0.009 (0.002) | 0.094 (0.019) | 0.096 (0.037) |
| Urinary bladder wall | 0.080 (0.019) | 0.077 (0.021) | 1.74 (0.54) | 1.37 (0.37) |
| Uterus/ Testes | 0.009 (0.002) | 0.020 (0.004) | 0.100 (0.016) | 0.174 (0.046) |
| Total body | 0.011 (0.002) | 0.011 (0.002) | 0.115 (0.016) | 0.126 (0.039) |

# **Supplemental figures**

## **Supplemental figure 1**


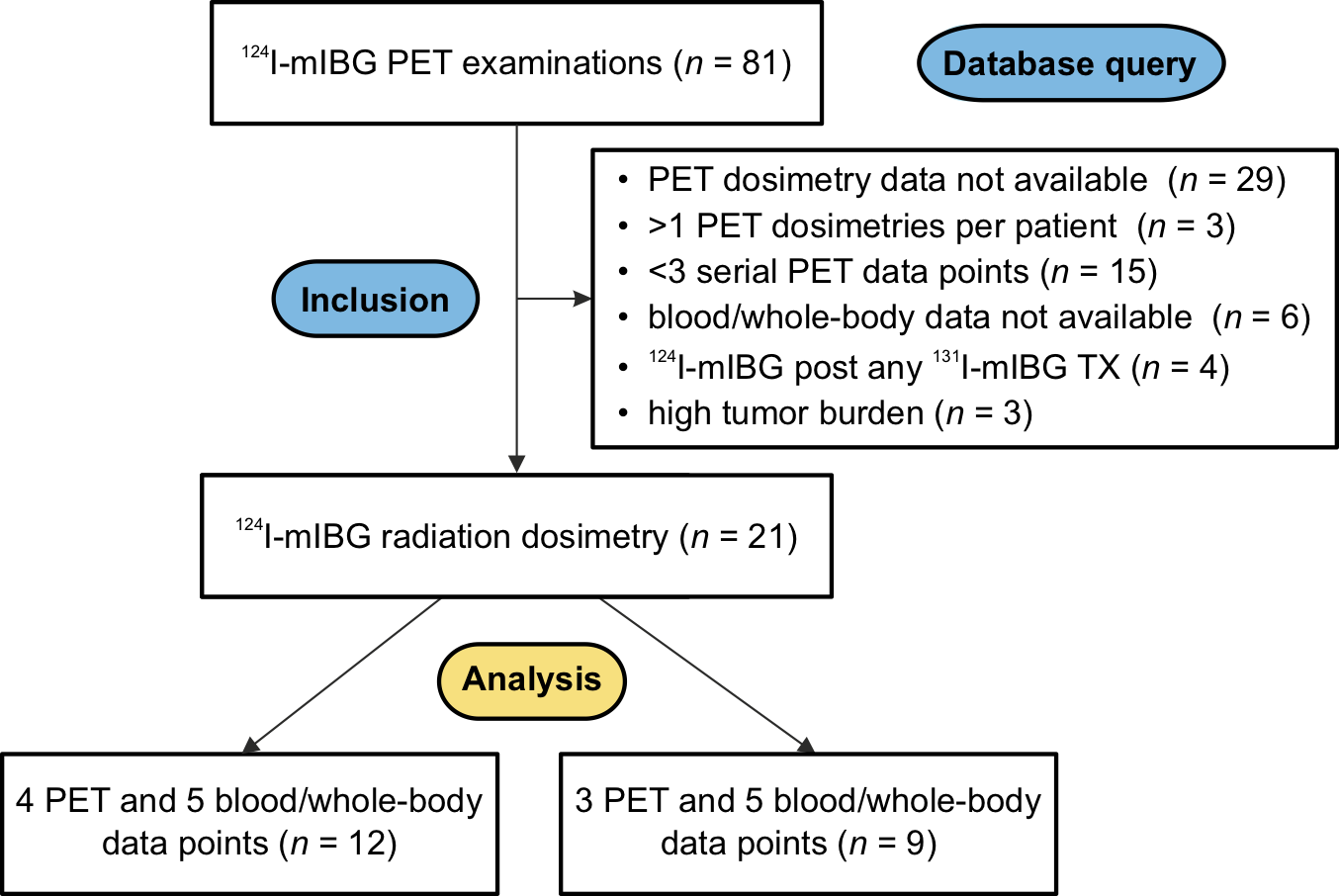


**Supplemental figure 1.** Flow chart showing who ^124^I-mIBG PET examinations in adult patients with neural crest tumours that were conducted at our institution (University Hospital Essen) between 2005 and 2015 and patients who were evaluated for radiation dosimetry according to the inclusion criteria. TX: therapy, blood data: blood sampling data, whole-body data: whole-body counting.

## **Supplemental figure 2**

| 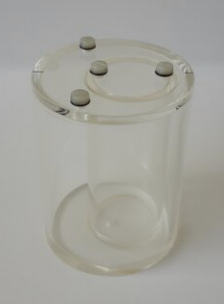 | 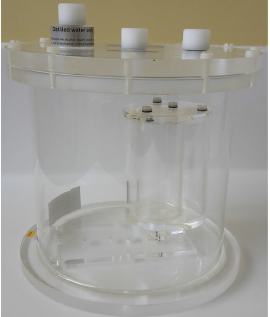 |
| --- | --- |
| 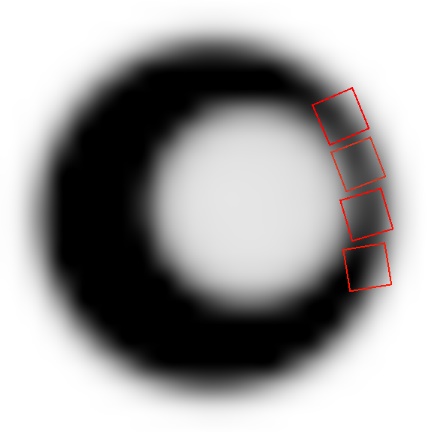 | 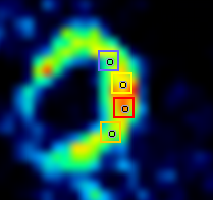 |
| Supplemental figure 2. PTW Heart phantom “C” (upper left) was mounted in a water-filled PTW Head phantom “B” (upper right). The PET images with four cubical VOIs of 10 mm side length are illustrated for the phantom (lower left) used to determine the heart wall recovery coefficient and a representative patient example (lower right). Phantom images were taken from the publication Tylski et al. (Tissue dose estimation after extravasation of 177Lu-DOTATATE, EJNMMI Physics 2021; 8:33). | |
